# ExIR enables prioritizing driver and biomarker genes from omics data in a reference free manner

**DOI:** 10.1016/j.isci.2026.116303

**Published:** 2026-06-08

**Authors:** Adrian Salavaty, Alon M. Douek, Jan Kaslin, Mirana Ramialison, Peter D. Currie

**Affiliations:** 1 Australian Regenerative Medicine Institute, Monash University, Clayton, VIC 3800, Australia; 2 Sir Peter MacCallum Department of Oncology, The University of Melbourne, Parkville, VIC 3010, Australia; 3 Division of Cancer Research, Peter MacCallum Cancer Centre, Melbourne, VIC, Australia; 4 Systems Biology Institute Australia, Monash University, Clayton, VIC 3800, Australia; 5 Novo Nordisk Foundation Centre for Stem Cell Medicine, Murdoch Children’s Research Institute, Royal Children’s Hospital, Flemington Road, Parkville, VIC 3052, Australia; 6 Department of Paediatrics, The Royal Children’s Hospital, University of Melbourne, Parkville, VIC 3052, Australia; 7 EMBL Australia, Monash University, Clayton, VIC 3800, Australia; 8 Institute of Biomedicine, Faculty of Medicine, University of Turku, Turku, Finland

**Keywords:** health sciences, medicine, bioinformatics

## Abstract

High-throughput sequencing enables genome-wide interrogation of biological systems, yet prioritizing functionally relevant genes and proteins from these data remains a key challenge. Here, we present ExIR (experimental data-based integrative ranking), a data-driven framework that classifies and ranks features as drivers, biomarkers, or mediators based on their behavior within inferred association networks. ExIR operates directly on experimental data without relying on external annotations. Across 14 transcriptomic and proteomic datasets, ExIR showed consistently strong performance in feature prioritization relative to commonly used methods. Application to RNA-seq data from a zebrafish model of mucopolysaccharidosis IIIA identified candidate regulators associated with disease progression. These results indicate that ExIR provides a generalizable approach for extracting biologically meaningful features from high-dimensional datasets, supporting more efficient downstream experimental investigation and interpretation.

## Highlights

• ExIR enables unbiased prioritization of drivers, biomarkers, and mediators
• ExIR is a standalone model requiring no external annotations or prior knowledge
• ExIR outperforms other methods in prioritizing driver, biomarker, and mediator genes
• ExIR analysis of zebrafish MPS IIIA transcriptomes reveals CNS biomarkers

## Graphical abstract

**Figure:**
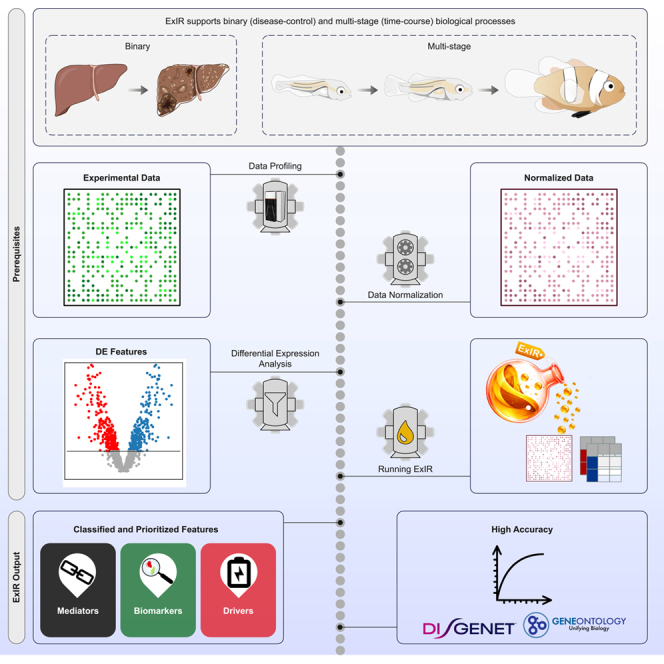

## Introduction

The emergence of advanced high-throughput technologies, including bulk and single-cell RNA sequencing as well as sophisticated mass spectrometry-based proteomic and metabolomic methodologies, have greatly enhanced the capacity for generating hypotheses relating to complex biological processes.^1^^,^^2^^,^^3^^,^^4^ Nevertheless, the subsequent experimental validation of these hypotheses invariably necessitates lower-throughput focused analytical approaches, including extensive manual curation and expert review of the generated data in the context of existing literature.^2^^,^^5^ Such approaches are heavily time-consuming, and the identification and selection for further investigation of the most relevant candidates from high-throughput datasets frequently represent the most significant bottleneck in progressing exploratory omics-based studies to verified biological findings.^2^^,^^5^

To address this bottleneck, a variety of computational models have been proposed to prioritize candidate features from lists of genes or proteins, such as those extracted from differential expression or abundance analyses. These prioritization algorithms employ a diverse range of strategies, including network-based algorithms and machine learning (ML) models, to filter and rank candidate features (e.g., genes and proteins). For example, graph-based frameworks have been developed to integrate multimodal data for enhanced prediction accuracy in related biological contexts such as gene regulatory networks,^6^^,^^7^ while diffusion models enable robust inference of cell-specific networks from noisy single-cell data.^8^ Similarly, feature selection techniques using recursive methods with random forests have improved classification of structural classes in low-similarity sequences, demonstrating the value of intrinsic data-driven prioritization.^9^ However, most of these models heavily rely on external—often manually curated and inherently incomplete—sources of evidence, such as gene ontology and pathway annotations, and protein-protein interactions (PPIs).^10^^,^^11^^,^^12^ Knowledge-based prioritization models manifest three significant shortcomings.

First, reliance on prior knowledge as an immutable input source often fails to account for the molecular heterogeneity inherent to a given biological sample.^13^ Second, the dependency on external information sources, particularly those assembled by text-mining of literature (e.g., co-mentioning of gene names) such as gene ontology (GO) and STRING databases,^14^ introduces bias favoring extensively researched genes,^15^ consequently elevating the risk of introducing both type I and II errors (i.e., false positive and false negative) during feature prioritization. Third, these models cannot be robustly applied in contexts where prior knowledge or supporting evidence sources is lacking; for example, in novel biological processes, or in molecular studies using non-model organisms. By contrast, a robust gene prioritization model solely reliant on the experimental data under investigation could circumvent these deficiencies, thereby augmenting accuracy of candidate feature prioritization. Approaches that detect sequence-level signatures from single genomes without annotated priors exemplify this potential, using statistical divergence measures and multiscale testing to identify atypical regions with high fidelity.^16^^,^^17^^,^^18^ Complementary methods that incorporate additional modalities like epigenetic signals, such as those that perform enhancer prioritization via ML on chromatin interactions, further highlight how data-intrinsic models can link regulatory elements to disease risk without external biases.^19^

In addition to the ranking of features to streamline experimental validation of high-throughput data, the ability to apply meaningful classifications to these features based on their inferred relevance to the underlying biological processes is of significant utility. While numerous models have emerged for the prioritization of genes, they largely overlook feature classification, including the crucial distinctions between the “driver,” “biomarker,” and “mediator” feature classification paradigms we introduce in this study (Box 1). Recent advances in inferring transcription factor activity from multimodal single-cell data offer promising avenues for such classifications, enabling prediction of drivers in cellular transitions or disease states by integrating regulatory networks with expression profiles.^20^^,^^21^^,^^22^^,^^23^ Here, “drivers” denote data-driven, process-level features inferred to influence biological state transitions, rather than genetically defined oncogenic driver mutations.

Box 1 **Key terminology of feature classification in ExIR**

**Drivers**

Drivers, such as genes or proteins depending on the contextual relevance, are ExIR network nodes whose expression or abundance is consistently and significantly altered in the same direction between conditions (e.g., across stages of a biological process or disease progression). In ExIR, the term “driver” refers to a data-driven, process-level feature that drives information flow through a network and is conceptually distinct from a driver mutation involved in oncogenic and other pathological processes.

Driver classification and ranking are not weighted by the magnitude of differential expression/abundance, but instead integrate statistical significance with global and local network influence. Driver nodes exert substantial influence on the flow of information across the ExIR network, which is taken to represent functional importance in the studied process. In a biological context, ExIR drivers represent features whose perturbation is predicted to accelerate or decelerate progression between experimental conditions, rather than asserting direct causal initiation of disease.

**Biomarkers**

Diagnostic biomarkers within the ExIR network are pivotal nodes—genes or proteins, depending on the experimental context—that exhibit significant and substantial changes in expression or abundance during biological processes or disease progression. Unlike drivers, which are assessed without consideration of the magnitude of change between conditions, biomarkers are scored based on the degree of their alterations. However, unlike drivers, the significance of genes/proteins in the information flow is not factored into biomarker scoring. Biomarkers are particularly adept at distinguishing between different biological conditions. A potential biomarker might also rank among the top drivers, and vice versa. In a biological context, biomarkers are characterized by their consistent expression changes across experimental conditions, although they may not directly contribute to driving the biological process or disease state under investigation.

**Mediators**

Depending on experimental design, mediator features may or may not be differentially expressed (DE).

**Non-DE mediators**

Non-DE mediators are nodes within the ExIR network that, despite not exhibiting differential expression/abundance in a pairwise comparison between experimental groups, are important nodes in directing the propagation of information throughout the network. Non-DE mediators are always first-/second-order neighbors of driver features, and are thus postulated to mediate the propagation of network information between influential driver nodes.

**DE mediators**

As with non-DE mediators, DE mediators are nodes that are first-/second-order neighbors of driver nodes in the ExIR network with a strong propensity for informational propagation in said network. Classification of a feature as a DE mediator requires that said feature fluctuates in its differential expression between groups, and thus detection of DE mediators necessitates >2 groups in the experimental design.

**Overlaps between driver and biomarker classifications**

Classification of a feature as a driver or biomarker in ExIR is not mutually exclusive; top-ranked drivers may also be top-ranked biomarkers (and vice versa). However, some DE/abundant features detected in a high-throughput experiment may reflect confounding factors (e.g., due to genomic or transcriptomic instability) rather than active drivers of a biological process.^24^^,^^25^ Thus, not all biomarkers are necessarily drivers.

**Mutual exclusivity of drivers and DE mediators**

In contrast to biomarkers and drivers, which can overlap, DE mediators and drivers are distinct by definition. Unlike drivers, which show consistent significant changes in the same direction across conditions in multi-condition/time point experiments, DE mediators exhibit fluctuating alterations between conditions. For instance, a DE mediator detected in a time-course experiment may be up-regulated from time point-1 to time point-2 but down-regulated from time point-2 to time point-3, and this fluctuation of directionality excludes its categorisation as a driver.

To address the above shortcomings, we developed ExIR (experimental data-based integrative ranking), a workflow that combines both supervised and unsupervised ML models as well as graph-based influence analyses to perform data-driven classification and prioritization of candidate features (e.g., genes, proteins, etc.). Uniquely, ExIR performs these tasks using solely the high-throughput experimental data under investigation (i.e., without reliance on external sources of knowledge). Benchmarking using several different transcriptomic and proteomic datasets demonstrates that ExIR outperforms other prioritization methods in specifically and sensitively identifying, classifying and ranking features by their functional importance to the biological process represented in a given dataset. Moreover, we demonstrate the utility of ExIR to decipher complex biological processes by applying the workflow to a transcriptomic dataset of a zebrafish model of the pediatric lysosomal storage disease MPS IIIA,^26^ facilitating identification of several mechanistic drivers of MPS IIIA disease progression as well as potentially clinically useful candidate diagnostic biomarkers.

## Results

### ExIR is a versatile data-driven framework for feature classification and prioritization

**Figure 1:**
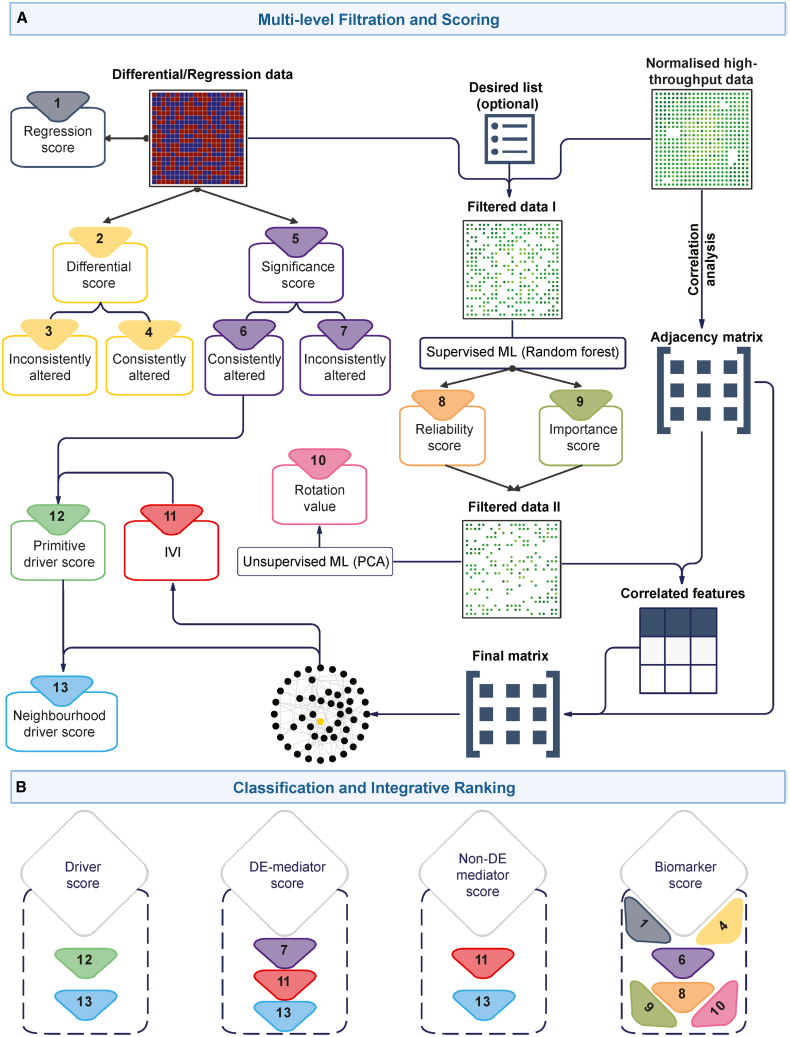
The ExIR model workflow (A) ExIR first performs multi-level filtration and scoring of input data (differential/regression data, normalized high-throughput data, and optionally a list of desired features). Differential/regression data are used for generation of multiple scores on the basis of their consistency of alteration between experimental groups to determine primitive and neighborhood driver scores. Filtered data (I) is subject to supervised ML through the Random Forest algorithm to derive reliability and importance scores, from which additional data filtration is performed (filtered data II). PCA-based unsupervised ML is then performed on this further filtered data to determine a rotation value. Simultaneously, an adjacency matrix is derived from the input normalized data by way of correlation analysis, and a tabulated set of correlated features is derived from intersection analysis of filtered data II. A final association matrix is generated by extending the adjacency matrix by one order using the set of correlated features. A connectivity network is then reconstructed from the final association matrix, which is used to calculate integrated values of influence (IVIs)^27^ for each feature within the network. IVIs and primitive driver scores are subsequently used to determine final neighborhood driver scores. (B) Combinations of scores derived from ExIR’s multi-level filtration and scoring pipeline define feature classifications and ranks. Drivers are classified based on their combined primitive and neighborhood driver scores; DE mediators (in datasets with >2 groups) are classified based on their IVIs and neighborhood driver scores and meeting the criteria of having an inconsistently altered significance score; non-DE mediators (not requiring experimental data with >2 groups) do not factor in significance scores. Biomarkers are defined by regression scores, consistently altered differential and significance scores, supervised ML-derived reliability and importance scores, and unsupervised ML-derived rotation values.

We developed ExIR, a data-centric framework that classifies and prioritizes biological features directly from high-throughput experimental data without reliance on external annotations or curated knowledge sources (Figure 1; STAR Methods). Starting from normalized input data, ExIR constructs feature-feature association networks using correlation-based adjacency matrices that capture global co-variation structure across samples. These adjacency matrices are subsequently used for network reconstruction and influence estimation. Supervised random forest modeling identifies features with predictive relevance to experimental conditions, while unsupervised principal component analysis captures dominant variance contributions independent of class labels. Together, these scores report feature influence and prioritization within the reconstructed network (Figure 1A; STAR Methods). ExIR integrates complementary supervised and unsupervised ML approaches with network-based influence analysis to derive multiple, independent feature-level scores capturing differential behavior, predictive importance, variance structure and network context. These data-derived scores are aggregated to classify features as drivers, biomarkers and mediators, followed by ranking within each class (Figure 1B; Table 1; STAR Methods). In this way, ExIR identifies features that function primarily as process-level drivers, diagnostic biomarkers, or network mediators, while also allowing biologically meaningful overlap between driver and biomarker roles.

**Table 1:** Conceptual definitions of drivers, biomarkers, and mediators in ExIR compared to conventional usage

Feature class	Conventional definition	ExIR definition	Key criteria in ExIR	What is new in ExIR
Drivers	genes whose genetic alterations (e.g., mutations, CNVs) causally initiate or sustain disease (especially cancer)	process-level driver features inferred from molecular data (e.g., transcriptomic/proteomic), defined by consistent perturbation and high network influence	(1) consistent and significant change across conditions; (2) high global and local network influence (IVI); (3) enriched driver neighborhood	decouples “driver” from mutation-centric definitions; identifies functional regulators of state transitions from data alone, independent of prior knowledge
Biomarkers	features that discriminate between biological states, often based on large and consistent changes in expression or abundance	features prioritized for discriminatory power, based on magnitude and statistical robustness of change, predictive importance, and variance contribution	(1) magnitude of differential expression/abundance; (2) statistical significance; (3) supervised importance; (4) variance contribution (PCA)	integrates multiple orthogonal criteria into a composite ranking, separating diagnostic signal from regulatory influence
Mediators	variables that lie on a causal path between an exposure and an outcome (typically defined via regression-based mediation analysis and effect size)	network-contextual features that facilitate information flow between driver-enriched regions, independent of a predefined causal chain	(1) high network influence (IVI); (2) strong driver-enriched neighborhood; (3) may be DE or non-DE; (4) not defined by pairwise causal models	moves beyond linear causal mediation to identify multi-node, network-level signal propagators in high-dimensional systems
Driver-biomarker overlap	typically not explicitly modeled	allowed; some features can be both drivers and biomarkers	shared satisfaction of both driver and biomarker criteria	explicitly separates regulatory importance vs. diagnostic utility, while allowing biologically meaningful overlap
Drivers vs. mediators	not formally distinguished in many frameworks	mutually exclusive (for DE mediators) based on expression behavior	drivers: consistent directional change; mediators: inconsistent or absent differential change	introduces a formal distinction between signal initiation and signal propagation roles

Side-by-side comparison of feature classes highlights their definitions, classification criteria, and biological interpretation in ExIR relative to conventional frameworks. The table emphasizes the key conceptual advances of ExIR, including data-driven identification of process-level drivers, multi-criteria biomarker prioritization, and network-based inference of mediators independent of predefined causal models.

Unlike other gene/protein prioritization methods such as Endeavour^28^ and ToppGene,^29^ which require a seed or training set to derive correlated features, ExIR relies only on interrogation of self-contained experimental data (Figure 1) for the detection of drivers, biomarkers, and mediators. The random forest algorithm prioritizes genes that would significantly affect inter-group variation between samples; ExIR then applies principal component analysis to weight features with a greater potential to differentiate all samples from each other, regardless of which group they belong to. Accordingly, those genes that have more sensitivity to inter-group variation and to phenotypic distinction within each group (i.e., intra-group variation) are assigned a higher rank.

### ExIR outperforms existing feature prioritization methods in driver ranking

**Figure 2:**
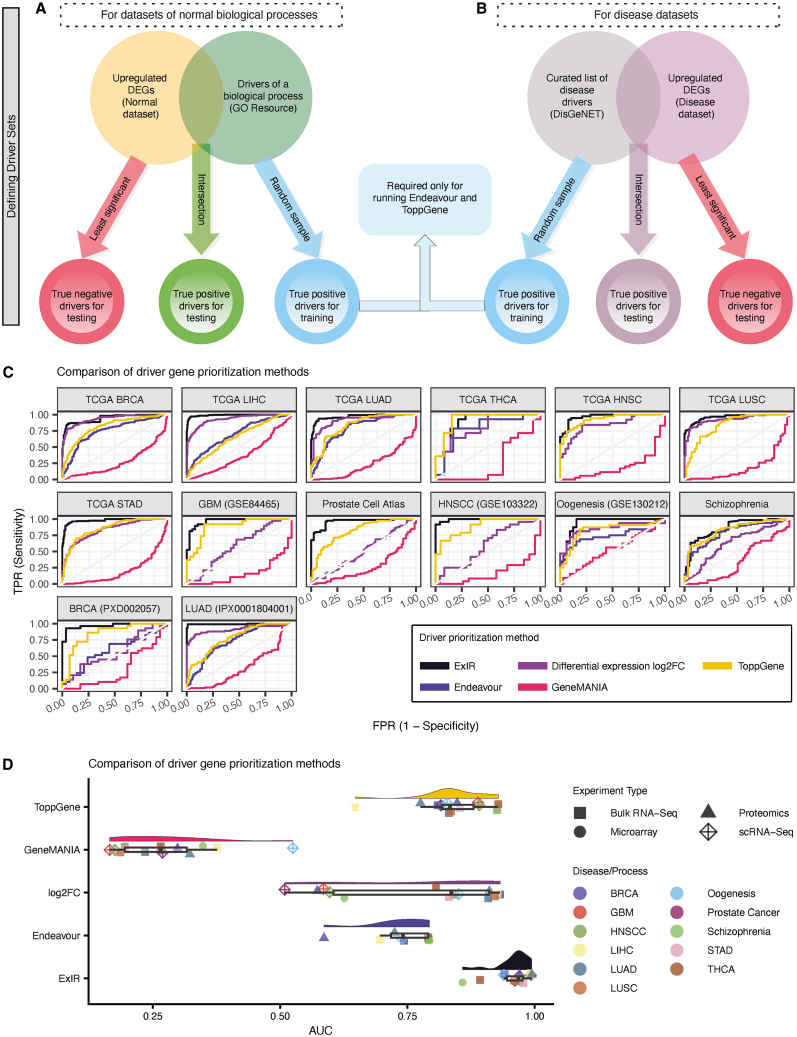
ExIR outperforms other common driver prioritization methods (A and B) Criteria used to define ground truth true positive and negative driver gene sets for benchmarking in homeostatic (A) and disease datasets (B). Note the requirement for a training set required for execution of Endeavor and ToppGene, which is not required for driver gene determination using ExIR. (C) ROC plots (true positive vs. false positive rates) of driver prioritization performance in TCGA BRCA, LIHC, LUAD, THCA, HNSC, STAD, LUSC, and GBM (GSE130212), Prostate Cell Atlas (EGAS00001005787), HNSCC (GSE103322), Schizophrenia (GSE93577), BRCA (PXD002057) and LUAD (IPX0001804001) datasets. (D) Raincloud plot summaries of ROC plot AUCs for comparison of performance of driver gene prioritization methods.

To examine ExIR’s performance in ranking influential driver genes/proteins against existing feature prioritization methods, we used a diverse set of published high-throughput transcriptomic and proteomic datasets representing several disease states (prostate cancer,^30^ head and neck squamous cell carcinoma [HNSCC],^31^^,^^32^ glioblastoma,^33^ schizophrenia,^34^ breast cancer,^32^^,^^35^ thyroid cancer,^32^ lung adenocarcinoma,^32^^,^^36^ stomach adenocarcinoma,^32^ lung squamous cell carcinoma,^32^ and hepatocellular carcinoma^32^). The use of pathology-associated data facilitates easier examination of model performance, as the transcriptional and proteomic changes associated with these pathological states are well-defined and provide a consistent ground truth. We additionally sought to examine ExIR’s performance outside of a disease context, and thus included a dataset representing a physiological process (oogenesis^37^). Collectively, the experimental modalities tested included bulk- and single-cell RNA sequencing, RNA microarray, as well as mass spectrometry-based proteomics datasets. In all cases, and to facilitate comparisons with methods that require training feature sets (i.e., Endeavor and ToppGene), benchmarking was performed by first assigning ground-truth (i.e., true positive and true negative) drivers on the basis of a consistent set of criteria from both gene expression/protein abundance data and ontological annotations (Figures 2A and 2B and STAR Methods).

Benchmarking using receiver operating characteristic (ROC) analyses demonstrated that ExIR consistently achieved the highest driver feature prioritization performance across datasets (Figure 2C). Across all prioritization benchmarks, ExIR attained a mean area under the ROC curve (AUC) of 0.96 (Figure 2D), exceeding the performance of ToppGene^29^ (mean AUC = 0.84), log_2_ fold change (0.77), Endeavour^28^ (0.73) and GeneMANIA^38^ (0.27). ExIR was the top-performing method in all datasets except TCGA THCA, where ToppGene marginally outperformed ExIR. These results indicate that ExIR provides robust and accurate prioritization across diverse experimental contexts while remaining independent of external knowledge sources. The low ROC performance observed for GeneMANIA reflects its reliance on external interaction networks and functional annotations,^38^ which are not optimized for prioritizing experimentally derived genes and proteins in this benchmarking context.

### ExIR improves biomarker gene prioritization relative to existing methods

**Figure 3:**
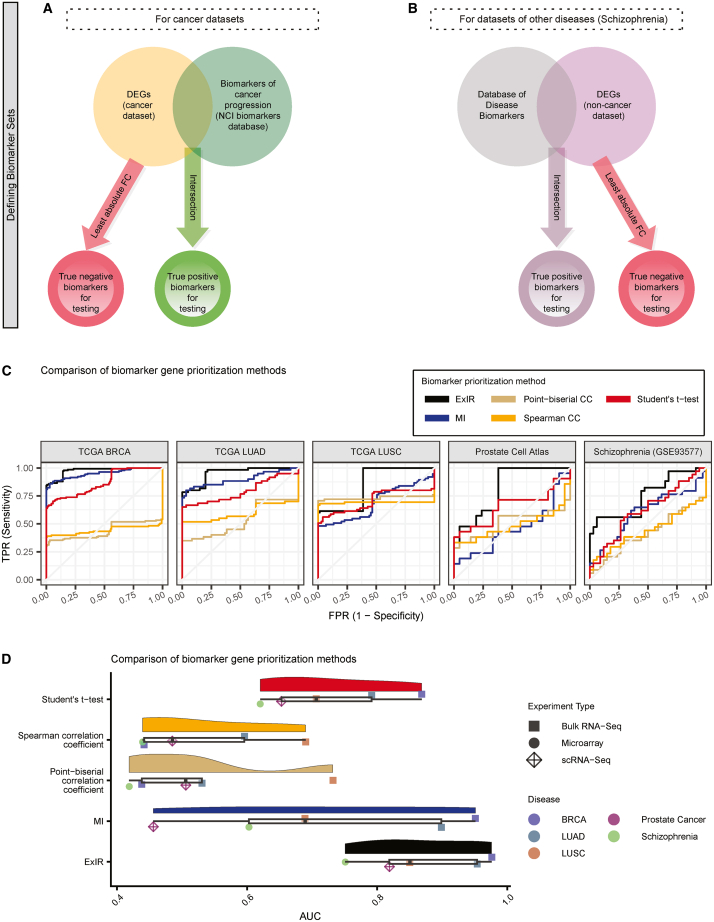
ExIR outperforms other common biomarker prioritization methods (A and B) Criteria used to define true positive and negative biomarkers from cancer datasets (A) and those of other diseases (B). (C) ROC plots (true positive vs. false positive rates) of biomarker prioritization performance in TCGA BRCA, LUAD, LUSC, and Prostate Cell Atlas (EGAS00001005787) and Schizophrenia (GSE93577) datasets. (D) Raincloud plot summaries of ROC plot AUCs for comparison of performance of biomarker gene prioritization methods.

Likewise, for biomarker gene prioritization, true positive and true negative biomarker sets were defined using a uniform set of criteria across both cancer and non-cancer disease datasets (Figures 3A and 3B and STAR Methods). ROC-based benchmarking demonstrated that ExIR consistently achieved superior performance in biomarker feature prioritization across datasets (Figure 3C). In particular, ExIR achieved a mean AUC of 0.87 (Figure 3D), outperforming Student’s *t* test (0.73), mutual information^39^ (MI) (0.72), and correlation-based approaches (0.53).

In addition to generally outperforming all other feature classification and ranking methods, our application of ExIR to the Cancer Genome Atlas lung adenocarcinoma RNA-sequencing dataset (TCGA LUAD) identified several biomarkers (Table 2), many of which fail to be detected as top-ranked biomarkers using other methods. Additionally, since all of the top five LUAD biomarkers prioritized by ExIR were down-regulated genes, we rationalized that detection of an up-regulated biomarker might represent a more traceable feature in a diagnostic context. We proceeded to assess the top five identified up-regulated biomarkers prioritized by ExIR; except for *SFTPC*, *PYCR1*, *TEDC2*, and *TOP2A* (each inconsistently prioritized by various methods), none of the other known or ExIR-predicted down-/up-regulated LUAD biomarkers were consistently identified as top-ranked biomarkers by methods benchmarked against ExIR (Table 2).

**Table 2:** Top known and ExIR-predicted LUAD biomarkers ranked by the benchmarked prioritization methods

Biomarker	Biomarker class	ExIR	MI	*t* test	PBCC	SCC
**Global Ranking: Known Biomarkers**						

*SFTPC*	known	1	3	3,079	14,960	14957
*SPP1*	known	170	603	1,918	5,515	167
*CBLC*	known	140	214	28	266	151
*MDK*	known	247	402	187	1,131	127
*MRC1*	known	471	539	6,821	14,718	14,724

**Global Ranking: Predicted Biomarkers**						

*AGER*	predicted	2	6	4,197	14,959	14,960
*EMP2*	predicted	3	40	3,859	14,949	14,945
*CAV1*	predicted	4	19	3,336	14,952	14,949
*RTKN2*	predicted	5	10	5,007	14,948	14,952

**Ranking within Up-regulated Biomarkers**						

*PYCR1*	predicted up-regulated	1	1	1	8	1
*TOP2A*	predicted up-regulated	3	14	83	639	5
*MMP11*	predicted up-regulated	4	164	3,604	6,947	48
*TEDC2 (C16orf59)*	predicted up-regulated	5	2	6	48	3
*IQGAP3*	predicted up-regulated	6	25	69	537	9

Top known and ExIR-predicted LUAD biomarkers (TCGA RNA-seq) ranked by ExIR versus benchmarked methods (MI, *t* test, PBCC, SCC), showing global rankings and separate rankings among up-regulated biomarkers. Lower numbers indicate higher priority.

**Figure 4:**
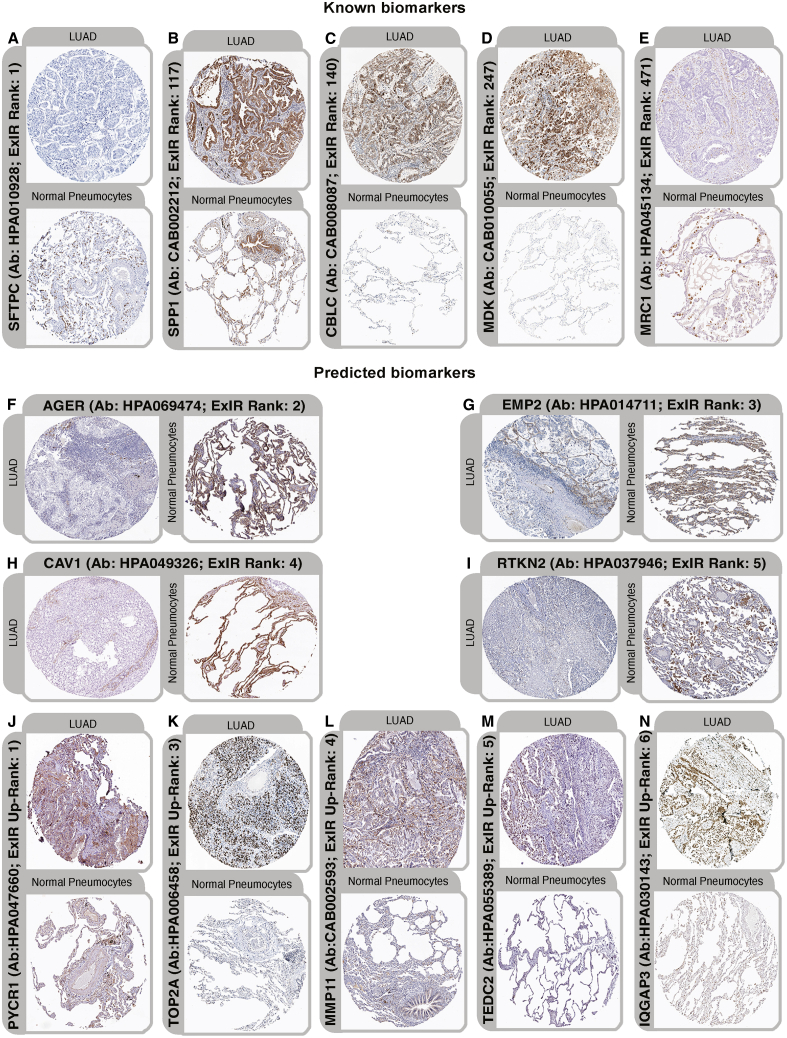
Differential immunoreactivity of known and novel ExIR-predicted LUAD biomarkers between normal and cancer samples (A–E) Immunohistochemical (IHC) data from the Human Protein Atlas^41^ database in LUAD and normal lung tissue for top five known LUAD biomarkers. (A) SFTPC–ExIR rank #1, LUAD (negative intensity; patient ID: 1847) and normal pneumocytes (quantity: 75%–25%; strong intensity; patient ID: 2268). (B) SPP1—ExIR rank #117, LUAD (quantity: >75%; moderate intensity; patient ID: 537) and normal pneumocytes (not detected; patient ID: 2268). (C) CBLC—ExIR rank #140, LUAD (quantity: >75%; moderate intensity; patient ID: 1847) and normal pneumocytes (not detected; patient ID: 2417). (D) MDK—ExIR rank #247, LUAD (quantity: 75%–25%; strong intensity; patient ID: 1847) and normal pneumocytes (not detected; patient ID: 2222). (E) MRC1 – ExIR rank #471, LUAD (undetected; patient ID: 1932) and normal macrophages (quantity: 75%–25%; strong intensity; patient ID: 2208). (F–I) IHC data of top five ExIR-predicted LUAD biomarkers (excluding SFTPC rank #1 already in a). (F) AGER – ExIR rank #2, LUAD (not detected; patient ID: 3144) and normal pneumocytes (quantity: >75%; strong intensity; patient ID: 4840). (G) EMP2 – ExIR rank #3, LUAD (not detected; patient ID: 1847) and normal pneumocytes (quantity: >75%; strong intensity; patient ID: 2101). (H) CAV1 – ExIR rank #4, LUAD (not detected; patient ID: 1249) and normal pneumocytes (quantity: >75%; strong intensity; patient ID: 2208). i, RTKN2 – ExIR rank 5, LUAD (not detected; patient ID: 3003) and normal pneumocytes (quantity: <25%; moderate intensity; patient ID: 2268).(J–N) IHC data of top six ExIR-predicted LUAD up-regulated biomarkers (excluding FAM83A rank #2, for which no IHC data was available). (J) PYCR1 – ExIR rank #1, LUAD (quantity: >75%; moderate intensity; patient ID: 2777) and normal pneumocytes (not detected; patient ID: 2208). (K) TOP2A – ExIR rank #3, LUAD (quantity: 75%–25%; strong intensity; patient ID: 3003) and normal pneumocytes (quantity: 75%–25%; weak intensity; patient ID: 2101). (L) MMP11 – ExIR rank #4, LUAD (quantity: >75%; weak intensity; patient ID: 1847) and normal pneumocytes (not detected; patient ID: 2438). (M) TEDC2– ExIR rank 5, LUAD (quantity: 75%–25%; moderate intensity; patient ID: 4208) and normal pneumocytes (not detected; patient ID: 1470). (N) IQGAP3– ExIR rank 6, LUAD (quantity: >75%; strong intensity; patient ID: 3048) and normal pneumocytes (not detected; patient ID: 1470). Per the Human Protein Atlas database usage guidelines, the link to the immunostaining images of all of the selected proteins in normal pneumocytes and LUAD samples are included as hyperlinks within the figure legend. Ab: antibody; LUAD: lung adenocarcinoma.

As the above biomarkers were retrieved from interrogation of an RNA sequencing dataset, and appreciating that quantified gene expression may not always accurately reflect protein abundance,^40^ we next sought to determine whether the ExIR-derived biomarker genes indeed evoke changes at the protein level in LUAD samples. Examination of public immunohistochemical data for top-ranked ExIR biomarkers in LUAD from the Human Protein Atlas^41^ revealed differential immunoreactivity of all previously established LUAD biomarkers between normal and cancer samples (Figures 4A–4E). Furthermore, the top five ExIR-predicted biomarkers (all down-regulated at the transcript level) exhibited pronounced immunoreactivity within healthy pneumocytes, yet were largely absent from tumor cells (Figures 4A–4F(i)). Subsequent examination of the top five ExIR-prioritized upregulated LUAD biomarkers likewise revealed differential immunoreactivity of these biomarkers between normal and LUAD samples (Figures 4J–4N). Collectively, this demonstrates ExIR’s capacity for sensitive identification of disease-associated biomarkers.

### ExIR identifies disease-associated driver and biomarker genes in a zebrafish model of MPS IIIA

**Figure 5:**
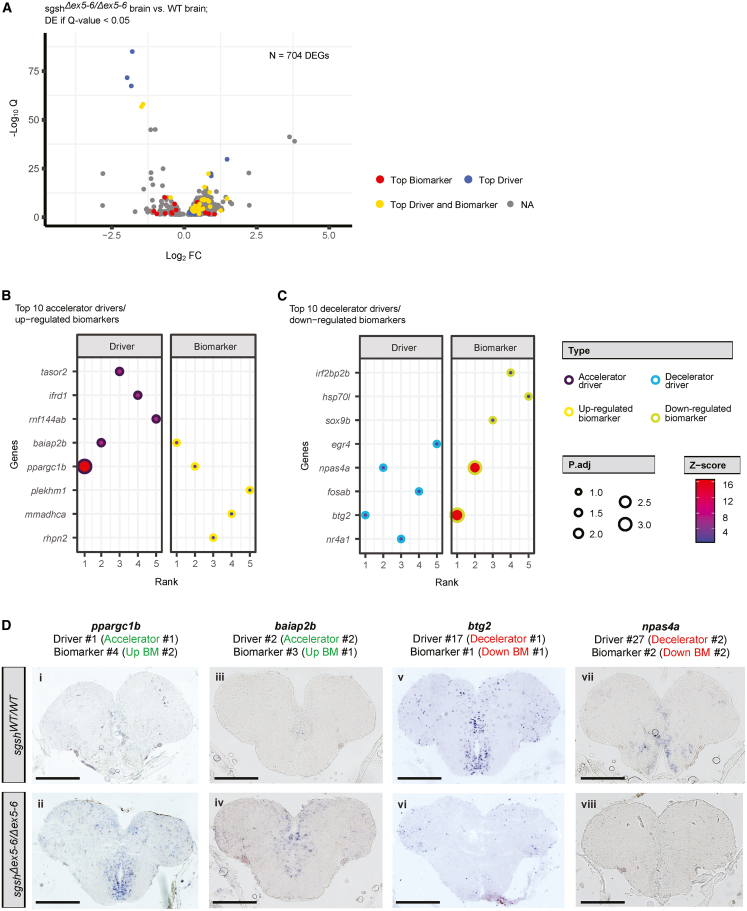
ExIR identifies novel brain-specific driver and biomarker genes associated with CNS disease in a zebrafish MPS IIIA model (A) Volcano plot of differentially-expressed genes (*Q* < 0.05, *n* = 704 genes) between 3-month-old wild type and *sgsh* brain demonstrating relationship between gene classification and classical DE analysis parameters. Points colored by ExIR gene type classification. (B) Top 10 ExIR-identified accelerating drivers/up-regulated biomarkers in the *sgsh* zebrafish brain. (C) Top 10 ExIR-identified decelerating drivers/down-regulated biomarkers in the *sgsh* zebrafish brain. (D) *In situ* hybridization analysis of top-ranked driver and biomarker genes in *sgsh* and wild-type sibling forebrain; i-ii, *ppargc1b*, iii-iv, *baiap2b*, v-vi, *btg2*, vii-viii, *npas4a*. Scale bars, 200 μm.

Mucopolysaccharidosis IIIA (MPS IIIA, or Sanfilippo syndrome A; OMIM #252900) is a lysosomal glycosaminoglycan storage disorder causing progressive neurological decline and onset of dementia-like symptoms in children, often leading to death during teenage years. Few effective therapeutic options are presently available for MPS IIIA patients; this is partially due to an incomplete understanding of the complex nature of MPS IIIA pathophysiology despite its monogenic etiology. To decipher the molecular features underlying MPS IIIA, we applied ExIR to a transcriptomic dataset of adult brains from the *sgsh*^*Δex5-6*^ zebrafish mutant^26^ (ZFIN line designation *sgsh*^*mnu301*^, hereafter referred to as *sgsh*), a zebrafish MPS IIIA model we previously generated. Interrogating the ExIR rankings for the 50 top-ranked driver and biomarker genes identified 67 distinct features; 33 genes were top-ranked in both classifications, with 17 genes each being top drivers but not biomarkers, and vice versa (Table S1). Examining the relationship between ExIR classification of differentially expressed genes (DEGs) and their differential expression levels demonstrated that identification of top drivers and biomarkers did not necessarily correlate with the DEGs exhibiting the largest fold-changes (Figure 5A). This further demonstrates that the network-based approach to gene classification and ranking is agnostic to basic parameters of differential gene expression analysis. Strikingly, of the 33 genes ranked as both top drivers and biomarkers detected in the transcriptome of *sgsh* brain, the vast majority of them clustered among the up-regulated DEG cohort (Figure 5A).

To further investigate the nature of ExIR-prioritized drivers and biomarkers, these features were next subdivided on the basis of fold-change directionality. Reflecting the above concordance between top drivers and biomarkers, the top two accelerating drivers—*ppargc1b* and *baiap2b* (a transcriptional coactivator reported to positively regulate mitochondrial biogenesis^42^ and an adapter protein involved in cytoskeletal reorganization of actin filaments,^43^ respectively)—were also the top two up-regulated biomarkers (Figure 5B), while the subsequent top-ranked accelerating drivers (*tasor2*, *ifrd1*, and *rnf144ab*) were also among the top-ranked up-regulated biomarkers (ranks 9, 6, and 8, respectively). Similarly, *rhpn2*, *mmadhca*, and *plekhm1* (up-regulated biomarker ranks 3, 4, and 5) were among the top-ranked accelerating drivers (8, 9, and 6, respectively; Table S2). This relationship was also observed, albeit to a lesser extent, among the decelerating drivers and down-regulated biomarkers. *btg2* and *npas4a* (both immediate-early genes [IEGs] associated with acute transcriptional responses to neuronal activity^44^) were both the top two decelerator drivers and down-regulated biomarkers (Figure 5C), though subsequent top-ranked decelerator drivers (*nr4a1*, *fosab*, and *egr4*, also well-known IEGs) tended to be lower-ranked down-regulated biomarkers (32, 33, and 34, respectively; Table S2). This is somewhat expected, given the observed bias in this dataset toward representation of up-regulated genes among top drivers and biomarkers (Figure 5A).

To further explore the nature of differential expression of the genes ranked as top drivers and biomarkers by ExIR in the *sgsh* zebrafish brain, we performed *in situ* hybridization against transcripts for the accelerating/up-regulated genes *ppargc1b* and *baiap2b*, and the decelerating/down-regulated genes *btg2* and *npas4a* in the telencephalon. As anticipated by its ranking as both a top up-regulated biomarker and accelerating driver in the *sgsh* CNS, *ppargc1b* was detectable in the *sgsh* telencephalon, but not that of wild-type siblings (Figure 5D(i and ii)); in the *sgsh* telencephalon, *ppargc1b* expression was strongest in the subventricular domain of the ventral subpallium (Figure 5D(ii)), with weaker expression in both the medial subventricular domain of the pallium and the parenchyma of the lateral pallium (Figure 5D(ii)). Similarly, little to no *baiap2b* expression was detected in the wild-type telencephalon (Figure 5D(iii)), while robust expression was observed in the *sgsh* telencephalon in the anterior parvocellular preoptic nucleus associated with the anterior-most hypothalamic region (Figure 5D(iv)), as well as the supracommissural nucleus of the ventral telencephalic area (Figure 5D(iv)).

Conversely, expression of down-regulated drivers and biomarkers *btg2* and *npas4a* were robustly detected in wild-type telencephalon, but were either reduced in a spatially distinct manner (in the case of *btg2*), or absent in expression (as with *npas4a*), in the *sgsh* brain. Wild-type expression of *btg2* was observed in abundant neurons in both dorsal, medial, and ventral subdivisions of the subventricular domain, with sparser expression in the posterior zone of the dorsal telencephalic area (Figure 5D(v)). Subventricular *btg2* expression was largely abolished in the *sgsh* telencephalon, though expression in the dorsolateral pallium was partially preserved (Figure 5D(vi)). Similarly, while *npas4a* expression was detected in all major subventricular domains of the wild-type telencephalon (Figure 5D(vii)), expression was effectively completely absent from these regions in homozygous *sgsh* siblings (Figure 5D(viii)).

Taken together, application of ExIR to a transcriptomic analysis of the brain of a zebrafish MPS IIIA model identified and prioritized features strongly characteristic of the disease state. That the abnormal expression patterns of all tested top-ranked genes were associated with specific neuronal domains suggests that certain neuronal subtypes or neuroanatomical regions may be variably susceptible to functional perturbation in MPS IIIA. By revealing specific neuronal domains susceptible to functional and molecular perturbations in MPS IIIA and prioritizing crucial features of the disease, our application of ExIR to this dataset opens avenues for deeper spatial and phenotypic exploration of the mechanisms underpinning MPS IIIA pathogenesis and progression.

### ExIR identifies genes mediating the transduction of driver signals

**Figure 6:**
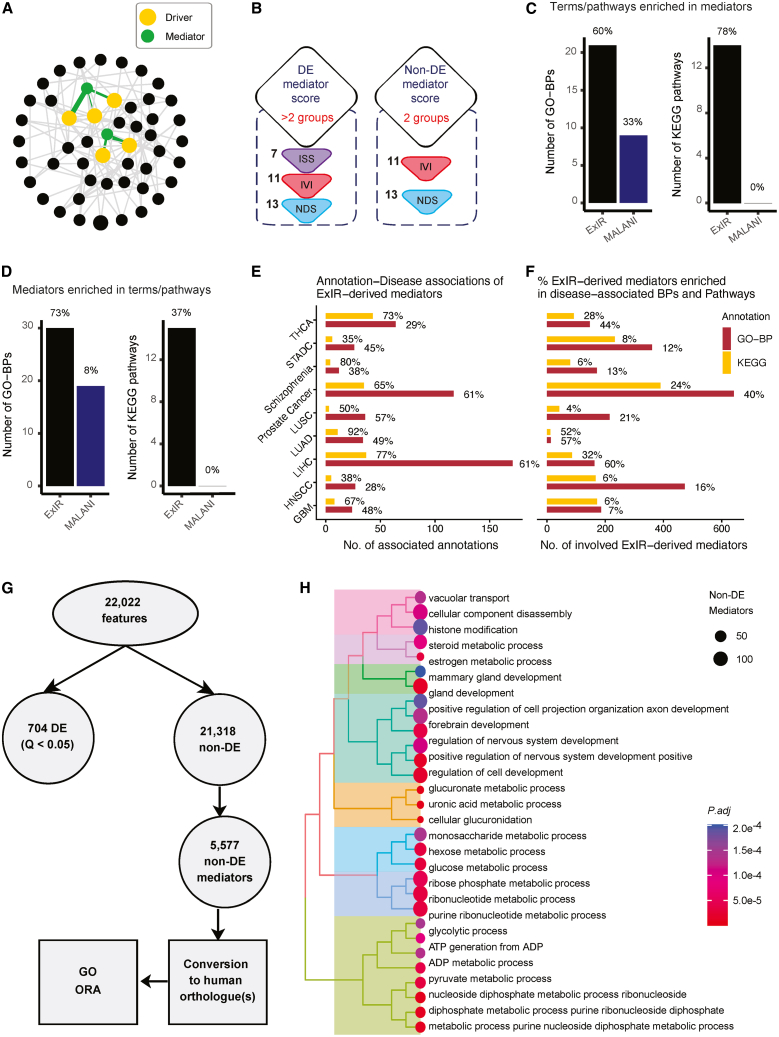
ExIR accurately classifies non-DE mediator genes functionally enriched for biological processes relevant to MPS IIIA pathobiology in the *sgsh* zebrafish brain transcriptome (A) Graphical representation of mediator relationship to drivers within a network. Edge weight represents strength of mediator association with first-order driver genes. (B) Differential ExIR score integration for DE- and non-DE mediator classification, dependent on experimental design. (C) Quantification of TCGA BRCA GO-BPs and KEGG pathways corresponding to ExIR- and MALANI-derived mediators. (D) Quantification of ExIR- and MALANI-derived mediators enriched in TCGA BRCA GO-BPs and KEGG pathways. (E) Number of associated GO-BP or KEGG pathway annotations associated with ExIR-derived mediators across different disease datasets. (F) Percentage of ExIR-derived mediators enriched in disease-associated GO-BPs and KEGG pathways. TCGA, The Cancer Genome Atlas; GBM, glioblastoma multiforme; BRCA, breast cancer; LIHC, liver hepatocellular carcinoma; LUAD, lung adenocarcinoma; THCA, thyroid cancer. (G) Flowchart diagram demonstrating refinement of mediators from the *sgsh* zebrafish brain transcriptome, followed by functional enrichment (overrepresentation, ORA) analysis. (H) Tree plot representation of significantly enriched gene ontology terms among ExIR-identified mediators associated with the *sgsh* zebrafish brain transcriptome.

An additional utility of ExIR is its capacity to assign features to a class we refer to as “mediators.” Mediators are features that are important for the flow of information between prioritized drivers in the ExIR-generated network (Figure 6A). Mediators may either be DE or not, depending on the nature of the experimental design employed (see Box 1). Most basically, mediators are never DE if an experiment contains only two comparator groups; detection of DE mediators requires an experimental design of >2 groups so as to fulfill the requirement for a given feature to be inconsistently DE (i.e., DE in some but not all pairwise comparisons within the dataset) and/or show opposing differential expression between different pairwise comparisons. Altogether, mediators exhibit either non-differential, or inconsistent/opposing differential expression/abundance patterns across multi-time point/multi-condition experiments (Figure 6B).

Mediators are predicted to directly and/or indirectly associate with major drivers of the biological process in question, inferred from their interactions with drivers within the ExIR network. However, they themselves do not necessitate a consistent differential expression/abundance pattern between conditions. Importantly, mediator prioritization reflects driver-contextualized network influence rather than nonspecific high-degree connectivity, distinguishing biologically meaningful signal transducers from dense correlation hubs. To our knowledge, only one other model (machine learning-assisted network inference, MALANI)^45^ has so far been described with the capacity to compute a class of genes akin (albeit not entirely identical) to our definition of mediator features. Additionally, MALANI has so far only been utilized in the context of transcriptomic analyses of cancer.^45^ To benchmark ExIR’s performance in detecting mediator features (which MALANI refers to as a “class II” gene), we applied ExIR to the same TCGA BRCA RNA-sequencing dataset as was previously interrogated with MALANI,^45^ and performed GO- and KEGG-based overrepresentation analyses on identified ExIR mediators/MALANI class II features. ExIR significantly outperformed MALANI in identification of mediators associated with breast cancer-related GO biological processes (GO-BPs) and KEGG pathways. The mediator-biological term association analysis was conducted using data from the Comparative Toxicogenomics Database.^46^ Extending this analysis to mediator identification across additional disease-related datasets revealed that, on average, more than 55% of GO-BP terms and KEGG pathways enriched among ExIR-derived mediators are implicated in the corresponding disease (Figures 6C–6F; Tables S3 and S4). Taken together, identification and classification of mediator nodes in the ExIR network allows for more thorough deconvolution of complex biological and pathological processes from high-throughput datasets.

### ExIR identifies potential non-DE transcriptional mediators in neuropathology of the MPS IIIA zebrafish

As all gene classes in ExIR—including mediators—are classified in an unbiased fashion, we sought to explore functional enrichment among the identified mediators in the ExIR network pertaining to the MPS IIIA zebrafish brain transcriptome. The MPS IIIA zebrafish RNA-seq dataset employed in this study comprised only two cohorts, and thus can only detect and rank non-DE mediators (Figure 6B). From the 22,022 genes detected in this RNA-seq dataset, 704 genes were defined as DE (in this case, differential expression is defined solely as Q < 0.05, with no log-fold change cut-off applied) and 21,318 non-DE features. Of the latter, ExIR identified 5,577 features as non-DE mediators based on their association with driver genes in the generated network (Figure 6G).

First, to account for limitations associated with zebrafish Gene Ontology annotations^47^^,^^48^^,^^49^ (compared to the more thoroughly curated human annotations), all zebrafish mediators were first converted to human orthologues, and the resulting gene set was then interrogated against the Gene Ontology resource.^50^ Strikingly, hierarchical clustering of significantly enriched (Q < 0.05, Benjamini-Hochberg *p* value adjustment) GO terms across all top-level ontology classes identified several GO term clusters of direct relevance to MPS IIIA pathology; these included terms associated with catabolism of the sugar/glycosaminoglycan substituents uronic acid and hexose. Further, among the significantly enriched terms were several associated with neural development and axonal projection (Figure 6H), likely relevant to the overt neuropathology characteristic of MPS IIIA. Collectively, this analysis demonstrates that ExIR is able to identify mediators which, despite their non-differential expression, are likely important lower-order regulators of identified drivers.

## Discussion

ExIR is a versatile model that simultaneously extracts, classifies, and prioritizes candidate features from high-throughput experimental data independent of external, curated knowledge sources. ExIR initially employs ML and graph-based influence evaluation techniques, then performs several filtration steps on the input normalized data to derive several different ranking scores. Combinations of these ranking scores are then used to derive summary scores for feature classification and ranking as drivers, biomarkers, and mediators of biological processes. From a biological perspective, manipulation of driver features prioritized by ExIR (potentially alongside co-manipulation of associated mediators) could have the most prominent impact on the progression of a biological process/disease as well as phenotype manifestation, thereby prioritizing candidates for therapeutic targeting. While highly ranked ExIR biomarkers are anticipated to have the highest sensitivity to a biological condition and the severity of the phenotype, their manipulation may not necessarily affect the progression of the process in the way highly ranked driver features might.

Our comparative analyses show that ExIR robustly outperforms existing tools and algorithms in the unbiased prioritization of ground truth driver, biomarker, and mediator features across diverse datasets representing multiple distinct experimental modalities. The underlying reasons for the superior accuracy and robustness of the ExIR model include (1) co-implementation of both supervised and unsupervised ML techniques; (2) integration of both ML and network-based models; (3) optimization of mathematical operations for score integration; and (4) independence from potentially-confounding external sources of information.

In order to experimentally validate ExIR’s utility in deciphering complex disease states, we employed this workflow to a transcriptomic analysis of the first zebrafish model of the mucopolysaccharidosis Sanfilippo syndrome Type A (MPS IIIA).^26^ The etiology of mucopolysaccharidoses, specifically the deficiency in glycosaminoglycan (GAG) catabolism, particularly heparan sulfate (HS) in the case of MPS IIIA, has long been established. However, the precise mechanism by which this deficiency results in the distinctive progressive functional neurodegeneration observed in individuals with mucopolysaccharidoses remains elusive. Several studies have implicated multiple factors contributing to disease progression, including microgliosis and neuroinflammation,^26^^,^^51^^,^^52^ CNS atrophy,^53^ and more recently an increasing recognition of the role of functional synaptic impairments.^54^^,^^55^^,^^56^ However, interventions targeting these factors have generally not been sufficient to ameliorate the severity of MPS IIIA or halt its progression. A systems-level analysis of the MPS IIIA CNS provides an opportunity to perform an unbiased survey of the molecular changes associated with disease onset and progression, which in turn may lead to the rational development of more effective, evidence-guided therapies. However, such analyses inevitably produce large volumes of information that require manual curation and filtration for interpretability; this often represents a significant analytical bottleneck. ExIR, by classifying and ranking features in an unbiased manner, provides an effective means of feature prioritization which may aid in focusing research efforts toward the most important biological changes in a given assay. Indeed, application of ExIR to the MPS IIIA zebrafish transcriptome has provided an unbiased basis for deconvolution of the complex transcriptional signature of the disease state, facilitating refinement of distinct candidate features and pathways for further study of the most critical elements of MPS IIIA neuropathology.

Taken together, ExIR establishes a reference-free framework for the classification and prioritization of genes and proteins directly from experimental data, without reliance on external annotations or curated knowledge bases. By integrating network-based inference with data-driven ranking, ExIR provides a generalizable strategy applicable to bulk and single-cell transcriptomic as well as proteomic datasets. Notably, the introduction of a distinct class of “mediator” genes expands the analytical landscape beyond conventional driver and biomarker identification, enabling more nuanced interrogation of regulatory hierarchies underlying complex biological processes and disease progression.

As high-throughput technologies continue to generate increasingly complex and high-dimensional datasets, approaches that distil interpretable and experimentally actionable signals are essential for making optimal use of these data. By streamlining feature classification and prioritization, ExIR facilitates hypothesis generation and accelerates downstream validation efforts. Its standalone and annotation-independent design positions it for broad applicability across basic, translational and clinical research settings, including precision medicine initiatives and rare disease studies where robust identification of candidate drivers and biomarkers is critical.

### Limitations of the study

Despite ExIR’s performance and generality, it has several limitations. As a purely data-driven and unsupervised framework, its prioritization is inherently dependent on the quality, depth, and statistical power of the input data, with smaller sample sizes potentially limiting the robustness of correlation-based network inference. While ExIR incorporates adjustable correlation and mutual rank filtering strategies and integrates multiple independent evidence streams to mitigate spurious associations, unmodeled technical variation or batch effects may still influence results if not adequately controlled upstream. In its current implementation, ExIR operates on a single data modality at a time and therefore does not capture cross-modal regulatory relationships arising from concurrent transcriptomic, proteomic, or chromatin accessibility measurements. Extending ExIR to natively integrate multimodal data, including joint RNA-seq, proteomic, and ATAC-seq profiles, represents an important direction for future development and will enable more comprehensive inference of regulatory drivers and mediators underlying complex biological processes.

## Resource availability

### Lead contact

Further information and requests for resources should be directed to and will be fulfilled by the lead contact, Peter D. Currie (peter.currie@monash.edu).

### Materials availability

This study did not generate new unique reagents.

### Data and code availability

• All datasets analyzed in this study are publicly available. The Sanfilippo zebrafish RNA sequencing dataset generated in this study has been deposited in the NCBI Gene Expression Omnibus (GEO) and is publicly available as of the date of publication under accession number GSE304151. Additional datasets used include GBM scRNA-seq (GSE84465), oogenesis scRNA-seq (GSE130212), HNSCC scRNA-seq (GSE103322), schizophrenia microarray data (GSE93577), the Prostate Cell Atlas dataset (EGAS00001005787), TCGA datasets accessed via the Broad GDAC Firehose (https://gdac.broadinstitute.org), as well as proteomics datasets including the LUAD dataset (IPX0001804001, iProX), and the BRCA dataset (PXD002057, PRIDE/ProteomeXchange). Accession numbers are listed in the key resources table.
• All code used in this study is publicly available. The ExIR method is implemented in the influential R and Python packages and is accessible via GitHub (https://github.com/asalavaty/influential; https://github.com/asalavaty/python-influential), CRAN (https://cran.r-project.org/package=influential), and PyPI (https://pypi.org/project/influential/). Documentation and usage examples are available in the package vignettes.
• Any additional information required to reanalyze the data reported in this paper is available from the lead contact upon request.

## Acknowledgments

The authors would like to thank Lan Nguyen for their constructive feedback on the development of ExIR and proteomic data analysis, and Monash AquaCore, Monash Micro Imaging, and the Monash Genomics and Bioinformatics Platform facilities for their excellent support. The results shown in this study are in part based on data generated by the TCGA Research Network (http://cancergenome.nih.gov/). This work was supported by 10.13039/501100001779Monash University, a 10.13039/501100000925National Health and Medical Research Council (NHMRC) Fellowship (1136567) to P.D.C., an 10.13039/501100000925NHMRC grant (1180905) to M.R., an NHMRC grants (GNT2013305, GNT2037953) to J.K., 10.13039/501100002341Research Council of Finland and 10.13039/501100006306Sigrid Juselius Foundation grants to J.K., an incubator grant from the 10.13039/100013918Sanfilippo Children’s Foundation (Australia) and the Cure Sanfilippo Foundation (US) to J.K., a Translational grant from the 10.13039/100013918Sanfilippo Children’s Foundation (Australia), the Cure Sanfilippo Foundation (US), Fundacja Sanfilippo (Poland), Sanfilippo Initiative (Germany), and H.A.N.D.S (Portugal, Spain and France) to J.K. A.D. and A.S. are supported by 10.13039/100015539Australian Government Research Training Program (RTP) scholarships. The Australian Regenerative Medicine Institute is supported by grants from the 10.13039/100007222State Government of Victoria and the 10.13039/100015539Australian Government.

## Author contributions

A.S., A.D., J.K., M.R., and P.D.C. conceptualized the study; A.S. developed ExIR and performed *in silico* benchmarking and data analysis; A.D. performed all *in vivo* experiments and analysis; A.S. and A.D. wrote the manuscript with contributions from J.K., M.R., and P.D.C.

## Declaration of interests

The authors declare no competing interests.

## Declaration of generative AI and AI-assisted technologies in the writing process

During the preparation of this work, the authors used LLMs in order to improve readability and language. After using this tool, the authors reviewed and edited the content as needed and take full responsibility for the content of the published article.

## STAR★Methods

### Key resources table

**Table:** 

REAGENT or RESOURCE	SOURCE	IDENTIFIER
**Experimental models: Organisms/strains**		

Zebrafish: sgsh^Δex5-6^ (TU)	Monash University AquaCore facility (this paper); original strain description (Douek et al.^26^)	ZFIN: ZDB-GENO-220906-1 (*aka sgsh*^*mnu301*^)

**Biological samples**		

Zebrafish brain tissue (3-month-old adults; *n* = 3 per replicate, 3 independent replicates)	This paper	N/A

**Chemicals, peptides, and recombinant proteins**		

TRIzol	Invitrogen	Cat#15596
Paraformaldehyde (PFA)	Sigma-Aldrich	Cat#158127
Proteinase K	Roche	Cat#3115879001
DIG RNA Labeling Mix	Roche	Cat#11277073910
T7 RNA polymerase	NEB	Cat#M0251S
SP6 RNA polymerase	NEB	Cat#M0207S
AP-conjugated anti-DIG sheep Fab fragments	Roche	Cat#11093274910; RRID: AB_514497
NBT-BCIP stock solution	Roche	Cat#11681451001

**Critical commercial assays**		

Agilent RNA 6000 Nano Kit	Agilent Technologies	Cat#5067-1511

**Deposited data**		

Sanfilippo zebrafish RNA-seq data	NCBI Gene Expression Omnibus (this paper)	GEO: GSE304151
GBM scRNA-seq dataset	GEO	GEO: GSE84465
Prostate Cell Atlas scRNA-seq dataset	EGA	EGA: EGAS00001005787
HNSCC scRNA-seq dataset	GEO	GEO: GSE103322
Oogenesis scRNA-seq dataset	GEO	GEO: GSE130212
Schizophrenia microarray dataset	GEO	GEO: GSE93577
TCGA datasets	Broad GDAC Firehose	https://gdac.broadinstitute.org
LUAD proteomics dataset	iProX	IPX0001804001
BRCA proteomics dataset	PRIDE (ProteomeXchange)	PXD002057

**Oligonucleotides**		

Primers for riboprobe cloning, see Table S9	This paper	N/A

**Recombinant DNA**		

pGEM-T-Easy vector	Promega	Cat#A1360

**Software and algorithms**		

R (v4.5.0)	R Core Team	https://www.r-project.org
ExIR	This paper	https://github.com/asalavaty/influential; https://cran.r-project.org/package=influential
influential R package (contains ExIR)	N/A	https://cran.r-project.org/package=influential
ranger R package	N/A	https://cran.r-project.org/package=ranger
igraph R package	N/A	https://igraph.org/r/
Seurat R package	N/A	https://satijalab.org/seurat/
TCGAbiolinks R package	N/A	https://bioconductor.org/packages/TCGAbiolinks/
GEOquery R package	N/A	https://bioconductor.org/packages/GEOquery/
limma R package	N/A	https://bioconductor.org/packages/limma/
DEP R package	N/A	https://bioconductor.org/packages/DEP/
plotROC R package	N/A	https://cran.r-project.org/package=plotROC
enrichR R package	N/A	https://cran.r-project.org/package=enrichR
clusterProfiler R package	N/A	https://bioconductor.org/packages/clusterProfiler/
EnhancedVolcano R package	N/A	https://bioconductor.org/packages/EnhancedVolcano/
DESeq2 R package	N/A	https://bioconductor.org/packages/DESeq2/
Bowtie2	N/A	http://bowtie-bio.sourceforge.net/bowtie2/index.shtml
Salmon	N/A	https://combine-lab.github.io/salmon/
HISAT2	N/A	http://daehwankimlab.github.io/hisat2/
SOAPnuke	N/A	https://github.com/BGI-flexlab/SOAPnuke

### Experimental model and study participant details

All protocols and procedures using zebrafish of ages greater than 7 dpf were approved by the Monash University Animal Ethics Committee (ERM14481, ERM22161 and ERM17993). Zebrafish were maintained under standard housing and breeding conditions^57^ in the AquaCore facility, Monash University. Embryos and larvae were maintained in E3 medium, while adult zebrafish were maintained in system water. Adult zebrafish (3-months-old) were randomly selected for inclusion in experiments without consideration of sex, which is not anticipated to be an important covariate in this study. Mutant strains used in this study were *sgsh*^*mnu301*^ (referred to either as *sgsh*^*Δex5-6*^ or simply *sgsh*).^26^

### Method details

#### Overview of the ExIR framework

ExIR is a data-driven computational framework designed to classify and prioritize biological features (for example, genes or proteins) as drivers, biomarkers, or mediators of biological processes directly from high-throughput experimental data. ExIR operates on normalized feature-by-sample matrices and associated differential or regression statistics derived from the same experiment. The framework comprises two conceptual stages: (i) extraction of multiple complementary feature-level scores capturing differential behavior, predictive importance, variance structure, and network influence; and (ii) integration of these scores using biologically-motivated additive and synergistic rules to classify and rank features according to their putative functional roles.

Unless otherwise stated, all computations were performed in R, using user-supplied, pre-normalized data.

#### Input data and preprocessing

ExIR requires two mandatory inputs. The first is a table of feature-level differential or regression statistics, such as log_2_ fold changes, regression coefficients, or associated statistical significance values derived from any standard differential expression or abundance analysis. In ExIR’s current form, these statistics may originate from bulk RNA sequencing, single-cell RNA sequencing, microarray, or proteomic assays. In multi-condition or time-course experiments, this table may contain statistics from multiple pairwise comparisons or regression analyses.

The second required input is a normalized feature-by-sample matrix representing the complete experimental dataset, in which rows correspond to features (genes or proteins) and columns correspond to samples or cells. ExIR does not impose a specific normalization strategy; instead, users are expected to apply an appropriate normalization method upstream, consistent with the experimental modality. An optional log-transformation may be applied within ExIR if the input data are not already log-scaled. ExIR deliberately does not perform data normalization, batch correction or differential analysis internally, as these preprocessing steps are highly modality- and study-specific and typically require careful, context-dependent parameterization and quality control by the user. This design ensures that ExIR remains agnostic to experimental platform while avoiding the introduction of inappropriate preprocessing assumptions.

Optionally, users may provide a third input consisting of a custom list of features of interest. When supplied, this list is used to restrict downstream analyses to a predefined feature subset, provided that all listed features are present in the normalized data matrix.

#### Additive and synergistic score integration strategy

ExIR integrates heterogeneous sources of evidence using two complementary principles, additive integration and synergistic integration. These reflect distinct biological assumptions:

Additive integration is applied when combining multiple independent observations of the same type of evidence, such as differential statistics across contrasts, regression coefficients across conditions, or neighborhood-level influence scores. In this setting, additive integration captures the cumulative magnitude or consistency of evidence without assuming interaction effects. Examples include the calculation of differential scores across multiple comparisons, regression scores across ordered conditions, and neighborhood driver scores computed as the sum of primitive driver scores of all first-order network neighbors. In experiments with a single comparison (for example, two-condition, single–time point studies), additive integration is omitted where not applicable.

Synergistic integration is applied when combining distinct, biologically orthogonal evidence streams for the same feature, under the assumption that concordant signals jointly strengthen functional relevance. In these cases, scores are combined multiplicatively to prioritize features that are simultaneously significant, influential, and predictive, while penalizing features supported by only a single evidence source. Synergistic integration is used in the calculation of primitive driver scores, final driver scores, biomarker scores, and both differentially expressed and non-differentially expressed mediator scores, as described below.

#### Calculation of differential, regression, and significance scores

For each feature, ExIR computes summary scores that quantify the magnitude, consistency, and statistical confidence of differential behavior across experimental conditions.

For experiments with two or more conditions, a differential score is calculated for each feature by summing the absolute values of user-supplied differential statistics (for example, log_2_ fold changes) across all relevant comparisons:

$${\text{Differential}}_{\text{score}}=\sum \left|{Differential}_{value}\right|$$

In multi-condition or time-course experiments, features are further subdivided into *consistently altered* and *inconsistently altered* categories based on whether the direction of change is preserved across comparisons. Separate differential scores are computed for each category.

For experiments involving ordered conditions or time points, an optional regression score is calculated for each feature by summing user-supplied regression coefficients or coefficients of determination (R^2^ values).

Statistical confidence is quantified using a significance score, computed as the sum of the negative log-transformed significance values (for example, adjusted P-values or false discovery rates):

$${\text{Significance}}_{\text{score}}=\sum -\log_{10}\left({\text{Significance}}_{\text{value}}\right)$$

As with differential scores, significance scores are computed separately for consistently and inconsistently altered features in multi-condition designs.

#### Supervised machine learning for feature importance estimation

To estimate predictive importance, ExIR employs random forest classification,^58^ using sample-level labels (for example, disease versus control) as the response variable and feature expression or abundance values as predictors. Each sample constitutes one observation, and each feature constitutes one predictor variable.^59^

Random forest models were implemented using the *ranger* R package.^60^ The number of trees was set to a large default value (10,000) to ensure stable convergence of impurity-corrected feature importance estimates. Because ExIR relies on relative ranking and permutation-based significance rather than absolute importance magnitudes, prioritization outcomes are robust to reasonable variation in ensemble size once importance estimates have stabilised. The number of variables considered at each split set to the square root of the total number of features. Feature importance was quantified using impurity-corrected importance scores, yielding a single importance value per feature that reflects its contribution to accurate sample classification.

To assess the statistical significance of feature importance scores, ExIR applies a permutation-based approach, in which sample labels are permuted to generate null distributions of importance values.^61^ By default, 100 permutations are used, providing a balance between computational efficiency and robust P-value estimation. P-values are calculated following the method of Altmann et al.*,*^62^ and transformed into a supervised-learning significance score using negative log_10_ transformation.

Following supervised learning, features may be further filtered based on statistical significance thresholds, reducing noise in downstream analyses.

#### Unsupervised machine learning for variance-driven feature prioritization

To capture unsupervised variation in the data, ExIR performs principal component analysis (PCA) on the filtered, normalized feature-by-sample matrix. PCA is conducted using base R functions.

For each feature, ExIR extracts the absolute loading (rotation value) on the first principal component, which captures the largest proportion of variance across samples. Higher-order components were not considered to avoid introducing dataset-specific noise or technical variation. Features with high absolute rotation values are interpreted as strong contributors to sample separation along the dominant variance axis. These rotation values are used to increase the sensitivity of biomarker prioritization by identifying features that explain major sources of biological variation independently of class labels.

#### Construction of gene–gene association networks

To infer feature–feature associations, ExIR computes pairwise Spearman correlations between all features across samples using a matrix-based linear algebra formulation, resulting in a symmetric feature-by-feature correlation matrix. Correlations are computed across the entire dataset, independent of experimental conditions, to capture global association structure. Both positive and negative correlations were considered by thresholding on the absolute correlation coefficient. By default, associations with |r| ≥ 0.5 were retained, balancing network sparsity and biological interpretability; however, this threshold is user-adjustable and may be relaxed or tightened depending on sample size and expected regulatory density. As an alternative filtering strategy, ExIR supports mutual rank (MR)–based thresholding, in which associations are retained if their mutual rank falls below a user-defined threshold (default MR ≤ 20). MR filtering reduces the influence of indirect or unstable correlations and is particularly advantageous for large or heterogeneous datasets.

From this global matrix, a refined adjacency matrix is constructed by retaining correlations involving features present in the filtered dataset. First-order associated features are identified, and second-order associations are subsequently retrieved by iteratively filtering the global correlation matrix. This stepwise approach yields a final adjacency matrix comprising first- and second-order feature associations, enabling efficient network reconstruction while limiting computational complexity.

#### Network reconstruction and influence estimation

The final adjacency matrix is used to reconstruct a feature association network using the *igraph* R package. Within this network, the Integrated Value of Influence (IVI) is computed for each node using the *influential* R package (https://cran.r-project.org/package=influential).^27^ IVI integrates multiple centrality and connectivity measures into a single score that quantifies the overall influence of each feature within the network.

#### Driver score calculation

To identify candidate driver features, ExIR computes a primitive driver score for each feature by multiplying its IVI value by the significance score of consistently altered features. This score captures features that are both statistically perturbed and highly influential within the network.

To account for local network context, ExIR further computes a neighborhood driver score by summing the primitive driver scores of all first-order neighboring features. The final driver score is calculated as the product of the primitive and neighborhood driver scores, prioritizing features that are influential, consistently altered, and embedded within clusters of other influential features.^63^ Driver scores quantify the likelihood that a feature functionally contributes to progression between experimental conditions based on integrated statistical and network evidence, and do not imply direct genetic causality. Driver features are further subclassified as accelerator or decelerator drivers based on the direction of differential change.

#### Mediator score calculation

Mediator features are classified into differentially expressed (DE mediators and non-differentially expressed (non-DE) mediators. Mediator prioritization requires concurrent satisfaction of high IVI and driver-enriched neighborhood scores, ensuring that mediators arise from driver-contextualized network influence rather than from nonspecific high-degree connectivity in dense correlation structures. Also, because ExIR mediators are inferred from network influence and neighborhood context rather than from pairwise causal models, they are ranked by relative mediator scores rather than assigned a single mediation effect size.

DE mediators are features that exhibit inconsistent differential behavior across conditions but maintain high network influence and proximity to driver features. Their scores integrate significance scores of inconsistently altered features with IVI and neighborhood driver scores.

Non-DE mediators are features that do not show statistically significant differential expression but are prioritized based on their IVI values and neighborhood driver scores, reflecting potential regulatory or coordinating roles within the network.

#### Biomarker score calculation

Biomarker prioritization integrates multiple complementary criteria. For each feature, a biomarker score is computed by multiplying its differential score, regression score (if applicable), significance score, supervised learning importance and significance scores, and unsupervised PCA rotation value. This composite score prioritizes features that are consistently altered, statistically robust, predictive of sample class, and explanatory of major variance in the data. Biomarkers are subclassified as up- or down-regulated based on directionality.

#### Statistical scaling and multiple-testing correction

All derived scores are standardized using Z-score transformation.^64^ P-values are computed from Z-score distributions and adjusted for multiple testing using the Benjamini–Hochberg procedure to control the false discovery rate.

#### Data preparation for ExIR evaluation and benchmarking

For evaluation and benchmarking of ExIR, the following criteria were required for selection of datasets:

1. The dataset should correspond to a disease/biological process with >40 curated driver genes in the DisGeNET database or Gene Ontology resource;
2. The dataset should have >100 samples/cells;
3. The dataset should include at least 2 conditions (*e.g.,* diseased vs. unaffected, different time points);
4. The dataset should have a comparable number of samples/cells within each condition.

Ground-truth driver and biomarker sets were selected from context-specific, independent annotation resources (GO-BP for normal biological processes; DisGeNET/MutPanning for disease drivers; NCI EDRN and disease-specific biomarker databases for biomarkers), and were used exclusively for downstream evaluation rather than model construction, thereby minimizing annotation leakage and database-specific bias.

Accordingly, the following datasets were used to evaluate ExIR and demonstrate its applicability in the classification and prioritization of features from microarray, bulk and single-cell RNA-seq (scRNA-seq), and proteomics experimental modalities.

##### Glioblastoma (GBM) dataset

GSE84465 is a scRNA-seq dataset generated using plate-based protocols from four patients with confirmed cases of primary GBM and comprises 3,589 cells.^33^ Tissue samples for this dataset originated from either the tumor core or the peritumoural cortical space. Major classes of cells, including neoplastic and non-neoplastic cells, were identified using immunopanning^65^ and further confirmed by comparison with other single-cell and bulk RNA-seq data. The dataset was originally normalized based on the read counts generated by HTSeq^66^ and filtration of genes with very low counts. Amongst all cell types previously identified, we selected two major subsets of cells including periphery regular (non-neoplastic) cells (n=1,184 cells) as the ‘normal’ set and tumor neoplastic cells (n=1,029 cells) as the cancer set, and filtered out genes with zero counts across all selected cells for downstream analysis.

##### Prostate Cell Atlas dataset

EGAS00001005787 is a scRNA-seq dataset^30^ generated using the 10x Chromium Single Cell 3’ v2 protocol from 10 patients aged 50-72 undergoing image-guided biopsies for suspicion of prostate cancer and comprises 15,492 cells. Tissue samples for this dataset originated from paired cancer biopsies and adjacent normal prostate tissue. Major classes of cells, including epithelial, stromal, and immune cells, were identified through unbiased clustering and validated by flow cytometry and confocal imaging. The dataset was originally processed using CellRanger for alignment and UMI counting, with QC filtration removing cells with fewer than 200 or more than 2,500 genes, mitochondrial content exceeding 30%, and genes expressed in fewer than 3 cells. Amongst all cell types previously identified, we selected luminal epithelial cells including normal (n=3,078 cells) as the ‘normal’ set and tumor (n=5,088 cells) as the cancer set, and filtered out cells with fewer than 200 genes and genes expressed in fewer than 3 cells for downstream analysis.

##### HNSCC scRNA-seq dataset

GSE103322 is a scRNA-seq dataset^31^ generated using a modified SMART-Seq2 protocol from 18 patients with primary oral cavity HNSCC tumors (including five with matched lymph node metastases) and comprises 5,902 cells. Tissue samples for this dataset originated from primary tumors and matched lymph node metastases in treatment-naïve patients. Major classes of cells, including neoplastic (n=2,539) and non-neoplastic (n=3,363) cells, were identified by inferring large-scale chromosomal copy-number variations (CNVs) from averaged expression across chromosomal intervals, epithelial marker expression scoring, and global expression patterns via clustering.^31^ The dataset was originally processed by aligning reads to the hg19 human genome reference, quantifying expression as log2(TPM/10+1).^31^

##### Oogenesis dataset

GSE130212 is a scRNA-seq dataset generated using plate-based protocols from fetal mouse ovaries at three developmental stages (E12.5, E14.5 and E16.5), together encompassing 19,144 FACS-sorted high-quality murine female germ cells.^37^ The dataset was originally normalized and DEGs between all time-points were detected in Seurat.^67^

##### TCGA BRCA

TCGA BRCA is a bulk RNA-seq of breast cancer generated by the TCGA project. Here, only primary tumor (#1,095) and solid tissue normal (#113) samples were retrieved using the TCGAbiolinks R package.^68^ The raw RNA-seq data were pre-processed based on the Array-Array Intensity Correlation (AAIC) method in TCGAbiolinks with default parameters (r > 0.6). Processed data underwent quantile normalization using the default parameters of TCGAanalyze_Filtering function for downstream analyses. All other TCGA datasets used in this work were pre-processed and normalized according to the same methods and parameters applied to this dataset.

##### TCGA THCA

TCGA THCA is a bulk RNA-seq dataset of thyroid carcinoma generated by the TCGA project. Samples retrieved were primary tumor (#505) and solid tissue normal (#59).

##### TCGA LUAD

TCGA LUAD is a bulk RNA-seq dataset of lung adenocarcinoma generated by the TCGA project. Samples retrieved were primary tumor (#515) and solid tissue normal (#59).

##### TCGA LIHC

TCGA LIHC is a bulk RNA-seq dataset of liver hepatocellular carcinoma generated by the TCGA project. Samples retrieved were primary tumor (#371) and solid tissue normal (#50).

##### TCGA HNSC

TCGA HNSC is a bulk RNA-seq dataset of head & neck squamous cell carcinoma generated by the TCGA project. Samples retrieved were primary tumor (#520) and solid tissue normal (#44).

##### TCGA STAD

TCGA STAD is a bulk RNA-seq dataset of stomach adenocarcinoma generated by the TCGA project. Samples retrieved were primary tumor (#412) and solid tissue normal (#36).

##### TCGA LUSC

TCGA LUSC is a bulk RNA-seq dataset of lung squamous cell carcinoma generated by the TCGA project. Samples retrieved were primary tumor (#502) and solid tissue normal (#51).

##### Schizophrenia dataset

GSE93577 is a total RNA microarray dataset generated from dysfunctional dorsolateral prefrontal cortex layer 3 parvalbumin neurons in 36 matched pairs of schizophrenia and unaffected cases using the Affymetrix Human Genome U219 array.^34^ This dataset contains 141 samples, including 71 healthy and 70 schizophrenia samples. The raw microarray data were retrieved from the GEO database utilizing the GEOquery R package^69^ and log-transformed prior to downstream analyses.

##### LUAD proteomics dataset

IPX0001804001 is a proteomics dataset generated from primary LUAD samples with paired non-cancerous tumor-adjacent tissues from treatment-naive patients by means of high-performance liquid chromatography-mass spectrometry (HPLC-MS) and label-free quantification.^36^ This dataset contains 206 samples including 103 normal and 103 LUAD samples. The MaxQuant-based pre-processed data was retrieved from the Integrated Proteome Resource.^70^

##### BRCA proteomics dataset

PXD002057 is a proteomics dataset generated from human breast cancer cell lines SKBR3 and BT474 and their lapatinib-resistant derivative cells by means of nano-scale HPLC-MS and label-free quantification.^35^ This dataset contains 20 samples, including 10 benign and 10 malignant samples. The MaxQuant-based pre-processed data of this dataset was retrieved from the LFQ-Analyst website (https://bioinformatics.erc.monash.edu/apps/LFQ-Analyst/).

#### Definition of ground truth sets for benchmarking

Ground truth driver genes were obtained from DisGeNET, the Gene Ontology resource, and the MutPanning database, providing experimentally supported annotations. Ground truth non-driver genes were selected from statistically significant features with the least significant adjusted P-values. Ground truth biomarkers were obtained from the NCI Early Detection Research Network and a literature-derived biomarker database, while negative biomarkers were selected from differentially expressed features with minimal fold changes.

#### Differential expression/abundance analyses in ExIR benchmarking

##### scRNA-seq datasets

Differential expression analysis (DEA) was performed using the Seurat R package.^71^ scRNA-seq data were prepared and processed within the Seurat framework by creating a Seurat object for each sample via *CreateSeuratObject*, followed by data normalization using *NormalizeData* with the “LogNormalize” method and a scale factor of 10,000, where applicable. DEA was conducted via the *FindMarkers* function to identify differentially expressed genes (DEGs) between cancer and normal cells, employing the default Wilcoxon rank sum test for statistical comparisons. Subsequently, log2-transformed fold change values were used to classify up- and down-regulated genes, with DEGs filtered to retain only those with an adjusted P-value ≤ 0.05.

##### TCGA bulk RNA-seq datasets

All TCGA datasets used in this work underwent DEA using the TCGAbiolinks R package, which implements functions of edgeR.^72^ Specifically, a common negative binomial dispersion was first estimated across all genes, and a negative binomial log-linear model was fit to the read counts for each gene. Then, pair-wise tests for differential expression between the two groups were performed. All P-values were adjusted, and DEGs with *Padj* > 0.05 were filtered out.

##### Schizophrenia dataset

Microarray DEA was performed using the limma R package,^73^ where a linear model was first fit to the expression data of each probe, followed by computing the contrasts of the fitted models with moderated empirical Bayes statistics. DEGs with *Padj* > 0.1 were filtered out.

##### Proteomics datasets

Proteomics data were analyzed using the R package DEP.^74^ More precisely, initially each dataset was filtered for proteins that had a maximum of 20 percent missing values in at least one condition within each dataset. Next, the variance of each dataset was normalized followed by a missing value imputation using the “man” algorithm.^74^ Lastly, the differential abundance of proteins was calculated using limma, and P-values were adjusted using the Benjamini-Hochberg procedure. Differentially abundant proteins with *Padj* > 0.05 were filtered out. In the case of the LUAD proteomics dataset, the differentially abundant proteins with |*log*_*2*_*FC*| < 1 were filtered out to maintain the most prominent characteristics of the disease for downstream analyses and benchmarking.

#### Evaluation of driver prioritization performance in ExIR

To assess the performance of ExIR in driver gene prioritization, sets of curated disease/biological process-associated genes of the above datasets were retrieved from either DisGeNET v7 or the Gene Ontology resource^50^ and considered as the ground truth (Table S5). Additionally, the ground truth drivers of the TCGA datasets were complemented with the driver genes proposed by the MutPanning web server (http://www.cancer-genes.org/).^75^ Next, an intersection analysis was performed to identify common genes between the sets of ground truth driver genes and the sets of significantly up-regulated genes in the selected datasets. As genes with the most statistically significant differential expression are more likely to be driver genes,^76^ sets of significantly differentially expressed genes with the least significant adjusted *Padj* were selected as true negatives, with each negative set matched in size to its corresponding positive set, thereby ensuring balanced class labels for ROC analysis. Then, these sets of true positive and negative genes were combined as an input into ExIR as the desired lists of genes. Importantly, curated gene sets were used exclusively for benchmarking and did not inform feature prioritization within ExIR, which operates independently of external annotations. The outputs were compared with four commonly used gene prioritization methods; log_2_ fold change (log2FC), GeneMANIA,^38^ Endeavour,^28^ and ToppGene.^29^ Owing to the discontinuation and limited availability of the Endeavour platform at the time of manuscript revision, Endeavour could be evaluated for only 8 of the 14 benchmark datasets. The performance of ExIR and comparator methods in prioritizing driver genes was assessed using receiver operating characteristic (ROC) analyses implemented with the plotROC R package.^77^ For each method, all genes in a given dataset were ranked once using the full dataset, without partitioning into training and test sets, as the evaluation focuses on ranking accuracy rather than predictive generalization. Ground-truth driver and non-driver gene sets were used to define true positive and negative labels. The plotROC function systematically varies the ranking threshold to generate ROC curves, enabling quantitative comparison of prioritization performance across methods. To obtain the prioritized driver genes, default parameters were used to run the GeneMANIA and ToppGene models. For Endeavour, the gene ontologies, Reactome pathways^78^ and STRING PPIs^79^ were selected for building the models. Moreover, the training sets required to run the Endeavour and ToppGene models included all genes previously retrieved from the DisGeNET, MutPanning and Gene Ontology databases, except for those genes selected for testing the models (Figures 2A and 2B).

#### Evaluation of biomarker prioritization performance in ExIR

To evaluate the performance of ExIR in sensitively and specifically identifying biomarkers, lists of biomarkers for both cancer and non-cancer diseases were obtained from the NCI EDRN (https://edrn.nci.nih.gov/biomarkers) and a knowledge-based database of disease-related biomarkers^80^ (accessed August 19, 2020), respectively. The EDRN has proposed >20 biomarkers for four out of the nine cancer types corresponding to the above datasets – specifically, lung and breast cancer. The lists of protein/proteomic biomarkers were retrieved from the EDRN (Table S6). Additionally, a list of schizophrenia biomarkers was derived from the database of disease-related biomarkers^80^ (Table S6), which employs a knowledge-driven text-mining approach to extract biomarkers for a wide variety of diseases. The common features between these sets and the previously obtained lists of significantly differentially expressed genes/proteins were considered as true positive biomarkers; similarly, the same number of DEGs with the lowest absolute fold changes were selected as true negative biomarkers (Figures 2C and 2D). Importantly, the curated gene sets were used solely for benchmarking purposes and did not influence feature prioritization within ExIR, which functions independently of any external annotations. The combined true positive and negative lists were input into ExIR as the desired lists of genes, and the outputs were compared with four commonly used biomarker prioritization methods: mutual information (MI),^39^ Student’s t-test, the point-biserial correlation coefficient, and the Spearman correlation coefficient. The evaluation and comparison of biomarker prioritization methods were performed based on ROC analyses. MI was calculated between the expression profile of each gene and the binary (0,1) sample labels using the mpmi R package (https://cran.r-project.org/package=mpmi).^81^ Similarly, point-biserial and Spearman correlation coefficients, as well as the Student’s t-test, were computed between gene expression profiles and binary sample labels using the stats R package. Additionally, immunohistochemical data for the specific lung adenocarcinoma (LUAD) subtype analyzed were available from the Human Protein Atlas. We therefore examined protein-level expression of the top five ExIR-prioritized LUAD biomarkers—including both previously established biomarkers from the ground-truth set and ExIR-predicted candidates (considering all genes as well as the subset of upregulated genes)—in unaffected and LUAD tissues using this resource^41^ (http://www.proteinatlas.org). For this analysis, ExIR was applied to the full LUAD dataset without providing predefined true positive or negative gene sets.

#### Evaluation of mediator identification and prioritization performance in ExIR

As there presently exists no centralized resource containing validated sets of gene mediators of biological processes or diseases, the performance of ExIR in identifying and prioritizing mediators was evaluated based on functional annotation of ExIR outputs. Initially, the performance of ExIR was assessed in comparison to the mediator genes inferred by the MALANI algorithm.^45^ In the context of cancer datasets, MALANI proposes two classes of genes; one being genes frequently differentially expressed or mutated, and another being those genes that are not differentially expressed but may mediate the coordination of oncogenic signals between DE/mutated genes.^45^ A set of breast cancer mediator genes has been proposed based on application of MALANI to the TCGA BRCA dataset; thus, to compare the performance of ExIR against MALANI for mediator detection, the entire TCGA BRCA dataset was input to ExIR without prior provision of any desired gene list. Next, an overrepresentation analysis (ORA) of all ExIR- and MALANI-derived mediators for biological processes and KEGG pathways was performed using the enrichR R package,^82^ and statistically non-significant terms were filtered out (*P* < 0.05). The association of significant biological processes and KEGG pathways corresponding to ExIR- and MALANI-derived mediators in breast cancer were then separately interrogated using the Comparative Toxicogenomics Database (CTD, accessed October 19, 2021),^46^ a manually-curated repository for literature-based and computationally inferred associations between genes, phenotypes, diseases, etc. Additionally, this benchmarking workflow was then applied beyond breast cancer to all other examined disease datasets.

#### RNA sequencing

For each experimental replicate, total RNA was extracted from n=3 freshly-dissected 3-month-old zebrafish brains in TRIzol (Invitrogen, 15596). Three independent experimental replicates were used for bulk RNA sequencing, and all samples were assayed for RNA integrity on an Agilent 2100 Bioanalyzer using the Agilent RNA 6000 Nano Kit. 150 bp paired-end sequencing was performed by BGI (Hong Kong) using the DNBseq platform. SOAPnuke^83^ was used for adaptor removal and low-quality read filtration, and genome mapping was performed with HISAT2.^84^ Clean reads were mapped to the reference genome using Bowtie2,^85^ and gene-length scaled transcript abundance estimates were calculated with Salmon.^86^ DESeq2^87^ was used for differential gene expression analysis. Downstream visualizations for RNA-seq data were generated in R using EnhancedVolcano,^88^ ExIR (this paper) and clusterProfiler.^89^ DESeq2 differential expression analysis outputs are provided in Table S7, and GO enrichment analyses of RNA datasets are listed in Table S8.

#### *In situ* hybridization

Riboprobes for *in situ* hybridization were generated by cloning transcript-specific PCR products from zebrafish brain cDNA libraries into pGEM-T-Easy (Promega, A1360) using the TA-cloning method. Insert directionality was confirmed by either PCR and/or Sanger sequencing, and plasmids were linearised to facilitate *in vitro* transcription of antisense riboprobes using either SP6 or T7 RNA polymerase and DIG-RNA labelling mix (Roche, 11277073910). All primers used to clone riboprobe sequences are listed in Table S9.

Adult zebrafish were rapidly euthanized in an ice-water slurry, and exsanguinated on ice by severing major vessels posterior to the anal pore. Whole brains were dissected from the neurocranium in cold 1x phosphate-buffered saline (PBS) pH 7.4, and immediately transferred to 4% paraformaldehyde (PFA, Sigma, 158127) in PBS for overnight fixation at 4°C with gentle rocking. After fixation, brains were cryoprotected in a sucrose-EDTA solution (20% sucrose, 20% 0.5 M EDTA pH 8 in 1x PBS) overnight at 4°C, then cryo-embedded in a mixture of sucrose and fish gelatin as previously described.^90^ Brains were serially cryosectioned at 16 μm thickness using a Leica CS3050S cryostat.

For *in situ* hybridization, sections were pre-fixed with 4% PFA in PBS pH 7.4 for one hour, then washed twice with 1x PBS with 0.3% Triton X-100 (PBS-Tx 0.3%) for 20 minutes. 10 mg/mL Proteinase K (Roche, 3115879001) was diluted 1:500 in PBS-Tx 0.3%, and sections were digested at RT for five minutes. Sections were then quickly washed with PBS-Tx, and Proteinase K digestion was stopped by incubating sections with 4% PFA at RT for 10 minutes, followed by 2x 10-minute washes with PBS-Tx 0.3%. A hybridization chamber was assembled using a slide box, containing filter paper saturated with a hybridization chamber solution (5 mL 10x Salt solution [1.95 M NaCl, 89 mM Tris-HCl, 11 mM Tris base, 50 mM NaH_2_PO_4_·2H_2_O, 50 mM Na_2_HPO_4_ and 63.68 mM EDTA], 25 mL formamide and 20 mL ddH_2_O, and the hybridization chamber was preheated to 60°C in an incubator. Antisense riboprobes were diluted 1:200 in hybridization buffer (1 mg/mL Torula RNA, 50% formamide, 1x Salt solution, 10% dextran sulfate, 1x Denhardt’s buffer, in ddH_2_O), vortexed and denatured at 70°C for 10 minutes prior to addition to sections. Parafilm was used to mitigate probe evaporation during hybridization. Probe hybridization was carried out overnight at 60°C. Unbound/weakly-hybridized probe was then removed by varying stringency through washing with 1x SSC buffer and 50% formamide in ddH_2_O 1x 15 minutes, then 2x 30 minutes at 62°C, followed by 2x 30-minute washes at RT in MABT. Sections were blocked in 2% DIG blocking reagent (Roche, 11096176001) in MABT for two hours at RT, then incubated for four hours at RT in AP-conjugated anti-DIG sheep Fab fragments (Roche, 11093274910) diluted 1:2000 in 2% DIG blocking reagent in MABT. Sections were then washed 4x 20 minutes in MABT at RT, and equilibrated in staining buffer (0.1 M NaCl, 0.05 M MgCl_2_, 0.1 M Tris pH 9.5, 0.1% Tween-20, all in ddH_2_O) for 5 minutes. Chromogenic detection was then performed using NBT-BCIP stock solution (Roche, 11681451001) diluted 1:50 in staining buffer until sufficient signal was observed. Development was terminated by incubating sections in 4% PFA for 30 minutes, then washed 3x 10 minutes in PBS. Sections were mounted with 50% glycerol prior to imaging on a Zeiss Imager.Z2 slide-scanning microscope.

### Quantification and statistical analysis

All statistical analyses were performed in R (v4.5.0) as described in the STAR Methods. Differential expression/abundance testing was performed using the Wilcoxon rank-sum test (Seurat), edgeR, limma, or DESeq2, depending on the dataset and experimental modality. Multiple-testing correction was applied where applicable using the Benjamini–Hochberg procedure. Benchmarking of driver, biomarker, and mediator prioritization was evaluated by ROC analyses. All statistical details (test name, test statistic, exact P-values, effect sizes, sample sizes, and number of biological units) are provided in the STAR Methods.

## Supplemental information

Table S1. Commonalities of top 50 drivers and biomarkers of *mucopolysaccharidosis IIIA* prioritized by ExIR

Table S2. Top up-/down-regulated drivers and biomarkers of *mucopolysaccharidosis IIIA* prioritized by ExIR

Table S3. Biological processes and pathways significantly associated with mediators of investigated diseases from ExIR and MALANI models

Table S4. Disease-associated biological processes and pathways corresponding to ExIR- or MALANI-derived mediators

Table S5. Curated ground-truth driver gene sets used for benchmarking

Table S6. Curated ground-truth biomarker gene sets used for benchmarking

Table S7. DESeq2 differential expression analysis between wild-type and *sgsh* zebrafish brain

Table S8. GO analyses of transcriptomic mediators conserved across ExIR analyses of *sgsh* zebrafish brain

Table S9. Primer sequences used for riboprobe cloning
